# Expression of acetylcholinesterase 1 is associated with brood rearing status in the honey bee, *Apis mellifera*

**DOI:** 10.1038/srep39864

**Published:** 2017-01-03

**Authors:** Young Ho Kim, Ju Hyeon Kim, Kyungmun Kim, Si Hyeock Lee

**Affiliations:** 1 Department of Agricultural Biotechnology, Seoul National University, Seoul, Korea; 2 Department of Applied Biology, Kyungpook National University, Sangju, Korea; 3 Department of Veterinary and Animal Sciences, University of Massachusetts, Amherst, MA, USA; 4 Research Institute for Agriculture and Life Sciences, Seoul National University, Seoul, Korea

## Abstract

Acetylcholinesterase 1 (AmAChE1) of the honey bee, *Apis mellifera,* has been suggested to have non-neuronal functions. A systematic expression profiling of AmAChE1 over a year-long cycle on a monthly basis revealed that AmAChE1 was predominantly expressed in both head and abdomen during the winter months and was moderately expressed during the rainy summer months. Interestingly, AmAChE1 expression was inhibited when bees were stimulated for brood rearing by placing overwintering beehives in strawberry greenhouses with a pollen diet, whereas it resumed when the beehives were moved back to the cold field, thereby suppressing brood rearing. In early spring, pollen diet supplementation accelerated the induction of brood-rearing activity and the inhibition of AmAChE1 expression. When active beehives were placed in a screen tent in late spring, thereby artificially suppressing brood-rearing activity, AmAChE1 was highly expressed. In contrast, AmAChE1 expression was inhibited when beehives were allowed to restore brood rearing by removing the screen, supporting the hypothesis that brood rearing status is a main factor in the regulation of AmAChE1 expression. Since brood rearing status is influenced by various stress factors, including temperature and diet shortage, our finding discreetly suggests that AmAChE1 is likely involved in the stress response or stress management.

Acetylcholinesterase (AChE, EC 3.1.1.7) plays an important role in the cholinergic synapses and neuromuscular junctions of both invertebrates and vertebrates by regulating the level of the neurotransmitter acetylcholine (ACh)^1^. AChE has been widely studied in various insect pest species because it is also the target of organophosphorus (OP) and carbamate (CB) insecticides.

Only one locus of the gene encoding AChE has been identified in vertebrates, while two different *ace* loci (*ace1*, encoding AChE1, which is paralogous to *Drosophila ace*; *ace2*, encoding AChE2, which is orthologous to *Drosophila ace*) have been characterized in various insect species^2^, except for in Cyclorrhaphan flies^2^,^3^, including *Drosophila melanogaster*^4^ and *Musca domestica*^5^, in which the *ace1* locus has been lost during evolution. The expression level of AChE1 is much higher than that of AChE2 in most insect pests possessing both *ace* genes^6^,^7^,^8^,^9^,^10^. In addition, insects that are resistant to OP and CB insecticides possess point mutations in the *ace1* gene that are responsible for target site insensitivity^11^,^12^, suggesting that AChE1 is likely the major AChE involved in synaptic transmission in insects having both AChE1 and AChE2^10^,^13^. However, a more recent study revealed that AChE2 was predominantly expressed as the main catalytic enzyme in 33 insect species among 100 insects examined^2^. In particular, social or subsocial hymenopteran insects, including bees, wasps and ants, appeared to express AChE2 as the major synaptic enzyme, suggesting that the putative functional transition from AChE1 to AChE2 is a common event, particularly in Hymenopteran insects.

In addition to its neuronal function, AChE has been elucidated to play other non-neuronal roles, including neurite outgrowth, synapse formation^14^, glia activation modulation, tau phosphorylation^15^, learning/memory^16^,^17^ and xenobiotic defense^18^,^19^,^20^. In insects possessing two AChEs, a major catalytic enzyme is suggested to possess neuronal function, while a minor enzyme lacking high catalytic activity to play non-neuronal roles^19^,^21^. In fact, according to our previous study on the characterization of AChE in the Western honey bee, *Apis mellifera*^19^, the membrane-anchored AChE2 (AmAChE2) had most of catalytic activity, while the soluble AChE1 (AmAChE1) exhibited little activity. Furthermore, AmAChE2 was abundantly observed only in the central nervous system, including the head and ganglia, whereas AmAChE1 was predominantly expressed not only in the central nervous system but also in the peripheral nervous system/non-neuronal tissues. When an equimolar mixture of two AmAChEs was pre-incubated with OPs and CBs, the presence of AmAChE1 significantly reduced the inhibition of AmAChE2, suggesting that the soluble AmAChE1 functions as a bioscavenger^19^. Taken together, the results suggest that AmAChE2 is mainly involved in synaptic transmission as a neuronal enzyme, whereas AmAChE1 has various non-neuronal functions, including chemical defense against xenobiotics, in honey bees. From an evolutionary perspective, however, the physiological function of AmAChE1 does not seem to be limited to defense. In *Tribolium castaneum*, a minor catalytic enzyme (AChE2) has been revealed to play a non-cholinergic role in female reproduction, embryo development and offspring growth^21^,^22^, suggesting that non-neuronal AChE is likely involved in various physiological functions.

According to our preliminary observation, the AmAChE1 expression level varied significantly depending on the different collection times and conditions. In this study, therefore, we investigated the expression profiles of AmAChE1 over a year-long cycle to discover any abiotic and biotic factors regulating the AmAChE1 expression. In addition, bee colony environments were artificially changed to identify any correlation between the bee colony activity and the AmAChE1 expression pattern.

## Results

### Seasonal expression profiling of AChE1

**Figure 1:**
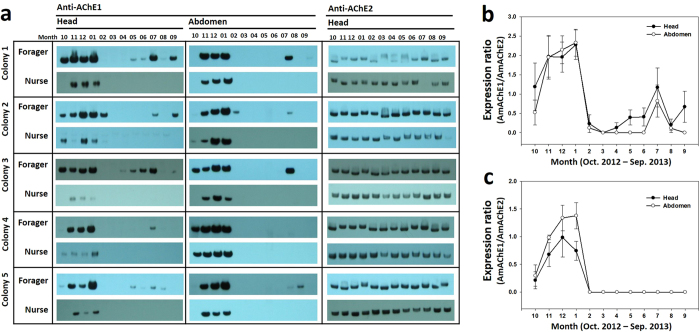
Seasonal expression profiles of AmAChE1 and AmAChE2 in honey bee. Honey bee samples were collected from 5 different colonies over one year. Protein samples extracted from the heads and abdomens of foragers and nurses were detected by Western blotting using anti-AmAChE1 and anti-AmAChE2 (**a**). The expression rates of AmAChE1 were normalized by calculating the expression ratio of AmAChE1 vs. AmAChE2 in foragers (**b**) and nurses (**c**).

We investigated the expression profiling of AmAChE1 and AmAChE2 over a year-long cycle by native polyacrylamide gel electrophoresis (PAGE) and Western blot analysis with AChE1- and AChE2-specific antibodies. Protein samples were prepared from the heads and abdomens of nurses and foragers that were collected every month. Based on our previous finding that AmAChE2 is not expressed in the abdomen while AmAChE1 is abundantly observed in both the head and abdomen^19^, protein samples from the head and abdomen were independently separated on a native PAGE gel for AmAChE1 detection, while only head protein was used for AmAChE2 detection (Fig. 1). As expected, AmAChE2 was consistently expressed throughout the year in both foragers and nurses, while the expression of AmAChE1 fluctuated with different seasons, indicating that AmAChE2 is a crucial enzyme for neurotransmission, as previously reported^19^. Therefore, we normalized the AmAChE1 expression rate by calculating the expression ratio of AmAChE1 vs. AmAChE2 (as an internal reference) based on the band intensity (Fig. 1b and c). As indicated by Western blot analysis (Fig. 1a), AmAChE1 was not detected in spring or fall but was significantly expressed during the winter months, with being moderately expressed during the rainy summer months in foragers. In particular, the expression level of AmAChE1 in foragers was more than two-fold higher than that of AmAChE2 in January (Fig. 1b). In nurses, AmAChE1 was also detected during the winter season with a similar band intensity as that of AmAChE2 but was not observed in the rainy summer months, indicating noticeable differences in expression pattern between the winter and spring-fall seasons (Fig. 1c). When the expression levels between the head and abdomen were compared, however, no marked differences were noticed in either forager (Fig. 1b) or nurse (Fig. 1c).

### The influence of environmental changes on the expression of AmAChE1

**Figure 2:**
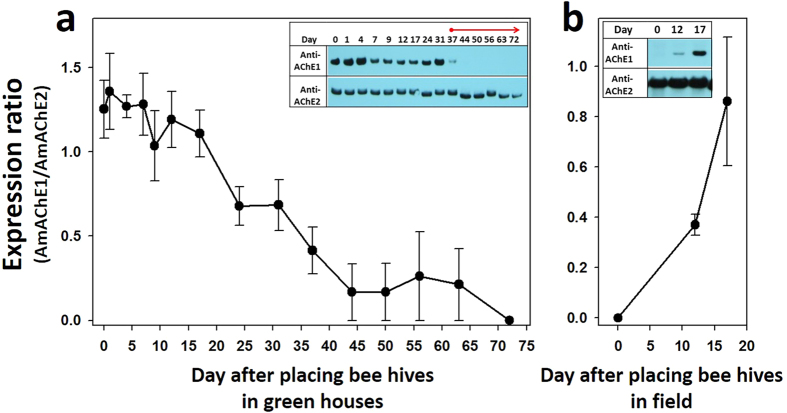
Changes in the AmAChE1 expression of overwintering honey bees following placement in a greenhouse (**a**) and field (**b**). Overwintering beehives were placed in a greenhouse for 72 days to resume brood rearing in winter (**a**). The beehives were moved back to the field to suppress brood rearing in winter (**b**). Two AChEs were detected by Western blotting (Fig. S2), and the expression rates of AmAChE1 were normalized by calculating the expression ratio of AmAChE1 vs. AmAChE2.

To eliminate possible environmental stress, thereby allowing bees to resume brood rearing in winter, four overwintering colonies were moved to strawberry greenhouses from a cold field. Because the seasonal expression pattern of AmAChE1 was similar between head and abdomen, as indicated in Fig. 1, only the heads were used for further studies. When bees were collected from the field in winter, AmAChE1 was strongly detected in all colonies (Figs 2a and S2a, see Day 0 lanes) as shown in its expression profiles in previous winter (Fig. 1). For three weeks, AmAChE1 was strongly detected in all colonies, and its expression began to diminish a month after placing overwintering beehives in greenhouses (Figs 2a and S2). During the first month in the greenhouse, the brood area was not stably extended, possibly due to a deficient diet, including pollen and honey, and low temperature (approx. 16 °C in average), despite the apparent foraging activity of bees inside the strawberry greenhouses. When artificial pollen diets were supplied to each hive on Day 37 following translocation, however, the brood area was steadily extended (field observation), and no AmAChE1 was observed after 2 weeks post-supplement of diet. According to the temperature record in the greenhouses (Fig. S2c), the temperature was not different between pre- and post-pollen diet supplement, strongly suggesting that nutritive conditions are essential for brood-rearing activity and closely related with the reduction of AmAChE1 expression. In contrast, when the four colonies were moved back to the field from greenhouses in February (the recorded temperature was approx. 1 °C), the brood-rearing activity was suppressed, and the brood area shrank briefly despite the presence of the pollen diet in the hive (field observation). In addition, no AmAChE1 was detected on the day when they were translocated to the field (Figs 2b and S2b, see Day 0 lanes), but AmAChE1 expression was strongly induced from 17 days post-exposure to cold weather (Figs 2b and S2).

**Figure 3:**
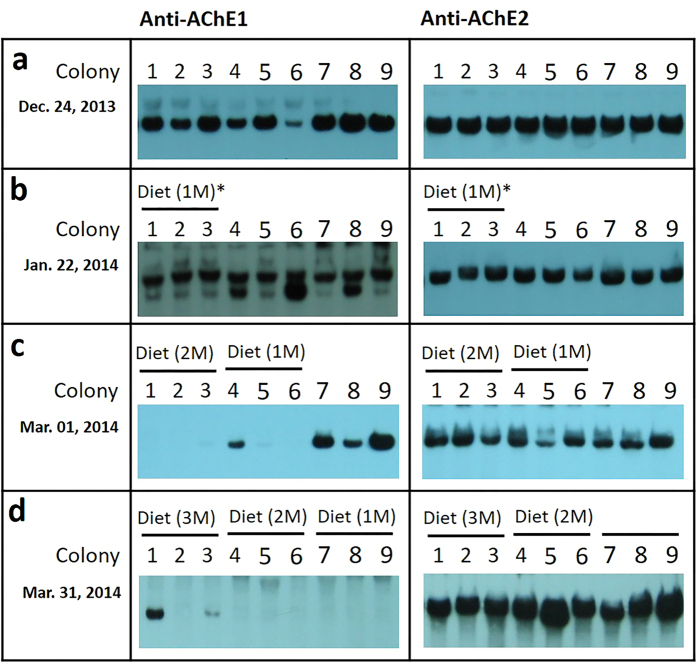
Induction of AmAChE1 expression in overwintering honey bee by supplementing with an artificial diet. Protein samples were prepared from nine overwintering beehives every month from Dec. 24, 2013 to Mar. 31, 2014. On Dec. 24, 2013, an artificial diet was initially supplied to Colonies 1, 2 and 3 (**a**). Colonies 1 to 6 and all of the colonies were supplied with the artificial diet on Jan. 22, 2014 (**b**) and Mar. 01, 2014 (**c**), respectively. A month later (on Mar. 31, 2014), protein samples, prepared every month, were separated in a native-PAGE gel, and two AChEs were detected by Western blotting. Asterisks indicate the period of artificial diet supplementation.

To investigate the effects of brood-rearing activity on AmAChE1 expression in a natural setting during winter, the brood rearing of overwintering colonies was stimulated by a sequentially supplying the pollen diet to the beehives at a one-month interval (Fig. 3). Before the diet was provided, AmAChE1 was strongly expressed in all 9 colonies (Fig. 3a), which was similar to the previous experiment (Fig. 1). When the pollen diet was supplied to Colonies 1–3 for a month (Dec. 24, 2013 – Jan. 22, 2014), the sealed brood area was not significantly extended (field observation) and AmAChE1 was still detected, although the brood-rearing activity was initiated (Fig. 3b). However, when Colonies 1–3 were further supplied with additional pollen diet, the brood-rearing activity became stronger and AmAChE1 expression was not detected (Fig. 3c). In Colonies 4–6, pollen diet was supplied one month later than in Colonies 1–3 (Jan. 22, 2014 – March 1, 2014), brood rearing began without delay, and AmAChE1 expression was not observed, except for in Colony 4 (Fig. 3c). These findings suggest that the initiation of brood rearing is regulated not only by a pollen diet but also by other seasonal factors (cold temperature, light-dark cycle, etc.). Nevertheless, the brood rearing status clearly correlated with AmAChE1 expression, which was similar to the AmAChE1 expression profiles observed in the greenhouse experiment (Fig. 2). Except for colony 4, the expression patterns of AmAChE1 were notably different between the honey bees supplied with a pollen diet for 1 or 2 months (Colonies 1 to 6) and those without diet (Colonies 7, 8 and 9) (Fig. 3c). Finally, all of the colonies except for Colony 1 exhibited no AmAChE1 expression when their brood rearing was artificially stimulated by a pollen diet at least for a month (for Colonies 7–9) (Fig. 3d). It is noteworthy that an additional fast-migrating band of AmAChE1 was observed in some minor cases (Fig. S2b and Fig. 3b), which appears to be generated by proteolytic cleavage as previous reported^19^. However, it remains to be elucidated how and why such an additional molecular form of AmAChE1 is generated.

**Figure 4:**
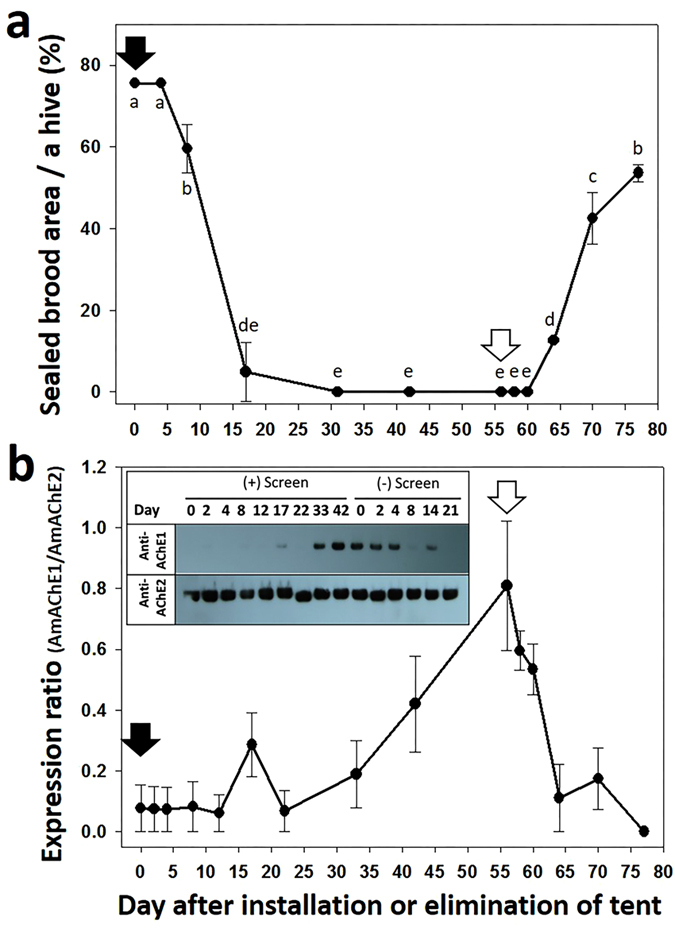
Suppression and subsequent restoration of brood rearing in active colonies by the installation and elimination of a screen tent, respectively, and changes in AmAChE1 expression. Four colonies exhibiting high brood-rearing activity were selected in late spring and placed in a screen tent to artificially suppress brood rearing by blocking foraging. At 56 days post-foraging inhibition, beehives were allowed to restore brood rearing by removing the screen. Enclosed and open arrows indicate the day of screen tent installation and elimination, respectively. During experiments, the sealed brood areas (**a**) and expression patterns of AmAChE1 (**b**) were measured. Different letters indicate the significantly different size of sealed brood areas (one-way ANOVA followed by Tukey’s test, P < 0.05) (**a**).

To confirm whether brood rearing status is closely associated with AmAChE1 expression in a different environmental setting, active beehives were placed in a screen tent, thus blocking normal foraging activity during the active brood-rearing season. Approximately 80% of a hive was covered with sealed brood on the day of the screen tent installation, and AmAChE1 was not detected in the head (Figs 4a and b and S4a). The sealed brood area rapidly shrank over time, and less than 10% of a hive was filled with sealed brood area on Day 17 post-screen installation. Brood rearing was completely suppressed until Day 56 (P < 0.05) (Fig. 4a). AmAChE1 was barely detected on Day 17 but was clearly detected on Days 42 and 56 following screen installation (Fig. 4b). Then, each colony was allowed to restore brood rearing by removing the screen. The sealed brood area steadily increased and reached a level covering approximately 60% of a hive (P < 0.05) (Fig. 4a). As brood rearing resumed, the expression of AmAChE1 was down-regulated, and no AmAChE1 was detected on Day 21 post-screen removal (Fig. 4b). A linear regression analysis also revealed that the sealed brood area significantly decreased after screen tent installation (P < 0.0001) but increased by removing the screen (P < 0.0001) (Table 1, Fig. S4b and c). The expression of AmAChE1 increased noticeably by the screen installation but decreased by the screen removal (Fig. S4d and e). These results confirm the inverse correlation between AmAChE1 expression and brood rearing status.

## Discussion

The seasonal AmAChE1 expression profile initially suggested that too high/low temperature, diet dearth and/or inhibition of foraging activity may be associated with AmAChE1 expression. In particular, when the temperature stress index was arbitrarily determined as the difference between the actual temperature and the reference temperature (16 °C), which is the threshold temperature for foraging^23^, the temperature stress index was reasonably correlated with the AmAChE1 expression level (Fig. S1c). Nevertheless, temperature stress was not the sole factor in regulating AmAChE1 expression, as seen both in the AmAChE1 expression during rainy summer months and in the greenhouse experiment, where the AmAChE1 expression pattern in overwintering bee colonies did not change until pollen diets were provided, even after being translocated to the greenhouse (average temperature was 16 °C) (Fig. 2a). AmAChE1 expression began to decrease only when brood rearing became active. In contrast, when the greenhouse colonies were moved back outside, the AmAChE1 expression level increased when the brood-rearing activity was significantly suppressed, even though sufficient pollen diets were present in the hives (Fig. 2b). Moreover, when tested with the normal healthy spring colonies without any temperature stress, where no AmAChE1 was expressed, the artificial inhibition of brood-rearing activity by screen tent installation induced the expression of AmAChE1, whereas its expression level rapidly decreased after the brood-rearing activity was restored by screen tent removal (Fig. 4). The field experiment during the winter season (Dec. 2013–Mar. 2014) also suggested that providing a pollen diet to overwintering colonies was not sufficient to suppress the expression of AmAChE1 (Fig. 3) unless brood rearing was fully activated by other environmental conditions. Taken together, it appears that any single factor of temperature stress or diet condition is not directly related to AmAChE1 expression, but both temperature and pollen diet are prerequisites for the determination of brood-rearing status, which is associated with AmAChE1 expression. In summary, through a systematic investigation of the AmAChE1 expression profiles in honey bee colonies with artificial stimulation or suppression of their brood-rearing activity, we found that high expression of AmAChE1 is observed in colonies not engaged in brood rearing, whereas no or little expression of AmAChE1 is found in a brood rearing-stimulating environment. The consistent observation of AmAChE1 expression during the rainy summer months, when brood rearing is known to be suppressed^24^, also supports this notion. However, it is unclear yet how AmAChE1 expression is associated with brood rearing status. AmAChE1 is likely involved in the signal pathways that are related to the brood-rearing activity, although it cannot be completely ruled out the possibility that the AmAChE1 expression level is a simple downstream phenomenon of the brood-rearing status. The high levels of AmAChE1 expression not only in the head (brain) but also in the abdomen (AmAChE1 expression was mostly observed in the fat body; Fig. S5) may suggest that the signaling pathway is not likely mediated by the neural pathway. In addition to its canonical role in synaptic transmission in the cholinergic nervous system, AChE is also known to be involved in various stress responses in mammals^25^,^26^,^27^,^28^,^29^,^30^. The generation of soluble AChE (read-through AChE, AChE-R) via alternative splicing is accompanied by various stresses, including oxidative damage, traumatic stress and disease in mammals^25^,^26^,^27^,^28^,^29^,^30^. A recent study also showed that the expression and kinetics of AChE were altered in response to low temperature stress in the silk worm silkworm *Philosamia ricini*^31^. Similarly, the expression of soluble AChE was also induced via alternative splicing following exposure to insecticide in *D. melanogaster*, which suggests its defensive role against xenobiotics^20^. Because soluble AmAChE1 is similar to *D. melanogaster* soluble AChE in molecular properties and is reported to function as a bioscavenger, AmAChE1 may be related to stress management in honey bee colonies, although the type of stress inducing the expression of AmAChE1 has not been accurately identified.

Cooperative brood rearing is a complex social behavior observed in eusocial insects and requires a tight control of sociality, such as labor division^32^,^33^, communication^17^,^34^, and stress management^35^,^36^. Assuming that brood rearing in a honey bee colony is normal behavior for sustaining the social community and maintaining nest homeostasis, perturbation of brood-rearing activity by sudden climate changes or some other reasons may result in stressful conditions. In the mammalian model, more ACh is released by nerve cells in the brain under mild stress conditions, in turn causing the overexpression of AChE-R to prevent the hyperexcitation of cholinergic circuits in both the CNS and PNS^27^. The overexpressed AChE-R is known to function as a stress signal indicating that the organism is under stress. If this is the case also for the honey bee, it is tempting to speculate that AmAChE1, which is likely a molecular homolog of the mammalian AChE-R, may also function as a stress signal molecule to initiate and regulate the stress management pathway. In fact, AmAChE1 was barely detected in nurses but strongly expressed in foragers during the rainy summer months (Fig. 1b). During the rainy summer month, foragers are expected to be placed in more stressful conditions as their foraging activity is completely blocked unlike nurses, of which in-hive environments are little different from those of dry seasons. Thus, the selective expression of AmAChE1 in foragers during the rainy summer seasons may support the idea that AmAChE1 is involved in stress management. The dissection of the honey bee stress regulation pathways using various stress markers at molecular, cellular and physiological levels would enable the elucidation of the possible association of AmAChE1 expression with various physiological outcomes of bees under different colony conditions.

## Materials and Methods

### Insects

Colonies of the Western honey bee (Italian hybrid) were mainly maintained in the field of the apiary in the Seoul National University experiment forest (Gwangju, Gyeonggi-do, Korea, 37° 18′ 43.2″N, 127° 18′ 40.8″E) in October and November 2012 and May to September 2013 and were overwintered from November 2012 to April 2013 at the beekeeping site in Goheung, Jeollanam-do, Korea (34° 30′ 38.0″N, 127° 19′00.8″E).

### Seasonal bee sampling

For the systematic expression profiling of AmAChE1 over a year-long cycle, ten different healthy colonies were selected, and approximately 20 foragers and nurses were collected monthly from each hive from Oct. 23, 2012 to Sep. 25, 2013. From spring to autumn, nurses and foragers were collected according to their ages and behaviors^17^,^32^: young worker bees found in the central region of brood comb were collected as nurses, whereas bees returning from their foraging trip or with pollen loads in their corbicula, especially in the foraging season, were collected as foragers. In winter, young bees in the central region and old bees in the peripheral region of the nest were collected as nurses and foragers, respectively, although all bees in winter showed the same physiological state^37^.

### Manipulation of brood-rearing activity

#### Stimulation and subsequent suppression of brood rearing in overwintering colonies (Greenhouse experiment)

To detect changes in the AmAChE1 expression patterns of overwintering bees when they resume brood rearing in the winter, four overwintering beehives maintained in the field apiary in Gwangju were selected, and each of them was placed in one of four individual strawberry greenhouses in December, 2013. On the 37th day after placement in the greenhouses, artificial pollen diets were supplied to each hive. After allowing for brood rearing by placing them in the greenhouse for 72 days, the beehives were moved back to the field apiary to suppress brood rearing. During experiments, the temperature was recorded in the two greenhouses and field using temperature data loggers (Hobo, Onset Computer Corp., Bourne, MA).

#### Stimulation of brood rearing in overwintering colonies by artificial pollen diet supply

To investigate the possibility of diet factor altering AmAChE1 expression, we supplied an artificial pollen diet directly to the beehives in winter. On December 24, 2013, we selected 9 overwintering bee colonies in Goheung, Jeollanam-do, Korea, and provided a pollen diet to Colonies 1, 2 and 3. A month later (on January 22, 2014), we collected honey bee samples from 9 colonies and provided a pollen diet to Colonies 1 to 6. On March 1, 2014, protein samples were prepared, and the diet was supplied to all of the colonies. Finally, on March 31, 2014, we extracted protein samples from the head from all colonies, and AmAChE1 and 2 were detected by Western blotting.

#### Suppression and subsequent restoration of brood rearing in active colonies (Screen tent experiment)

To suppress brood rearing by blocking foraging, four active beehives were placed in a screen tent for 42 days in late spring (from April to June) in 2014 until brood rearing was completely suppressed and then allowed to restore brood rearing by removing the screen. During the experiments, a brood-rearing comb was selected from each hive, and its sealed brood area was measured by a standard method^38^.

#### Protein sample preparation, native-PAGE and Western blotting

Protein samples were prepared from the head and abdomen of the collected honey bees by homogenizing with 0.1 M Tris-HCl (pH 7.8) containing 0.5% Triton X-100 and protease inhibitor cocktail (Invitrogen, Carlsbad, CA) using a bullet blender (Next advance, Averill Park, NY). The homogenates were centrifuged at 12,000 × g for 15 min at 4 °C. The supernatant was filtered twice through glass wool to remove excess lipid as previously described with some modifications^20^. The concentration of protein samples was determined by the Bradford method using bovine serum albumin as a standard protein^39^, followed by storage at −75 °C until further processing.

Polyclonal antibodies used for the detection of AmAChEs were generated as previously described^13^,^19^. For the synthesis of immunogenic peptides, two separate regions were chosen for each AChE (AChE1 peptide I and II vs. AChE2 peptide I and II) based on the highly conserved domains of AChEs across various insect species, including the honey bee (Fig. S6). The immunogenic peptides were synthesized according to the sequences of *B. germanica* AChE1 (QIIDTVFGDFPG for AChE1 peptide I and IIYYLTELFRKEE for AChE1 peptide II) or AChE2 (QERYEYFKGFEGEE for AChE2 peptide I and TYFILYDFMDYFEKD for AChE2 peptide II), where underlined sequences are identical to the corresponding amino acid sequences of the honey bee AmAChEs^19^. The amino acid sequences of AChE1 peptides I and II showed 69% and 75% sequence identities to the corresponding regions of AmAChE1, respectively. Likewise, the amino acid sequences of AChE2 peptides I and II exhibited 86% and 87% sequence identities to the corresponding regions of AmAChE2. Rabbits were immunized three times with 0.5 mg of each set of synthesized peptides (AChE1 peptides I and II or AChE2 peptides I and II) conjugated with bovine serum albumin (Ab Frontier, Seoul, Korea). Serum-specific antibodies were affinity-purified on columns using immobilized antigen peptides as described previously^19^. The resulting anti-AChE1 and anti-AChE antibodies were confirmed to exhibit high levels of specificity to respective AmAChE and little cross-reactivity between AmAChE1 and AmAChE2^2^,^19^.

Native-PAGE and Western blotting were carried out as previously described with some modifications^2^,^19^. Protein samples (8 μg) were separated on a native-PAGE gel (7.5%) in duplicate at 120 V for 90 min. After native-PAGE, the bands were transferred onto Hybond-N nitrocellulose membranes (GE Healthcare, Pittsburgh, PA) by electroblotting. Following blocking with blocking solution [PBS buffer containing 0.1% Tween-20 (PBST) and 5% fat-free milk] for 30 min at room temperature, two sets of nitrocellulose membrane sheets were incubated for 90 min at room temperature with respective polyclonal antibodies. The membranes were then rinsed three times with PBST buffer (each for 10 min). The rinsed membranes were incubated with horseradish peroxidase-conjugated anti-rabbit IgG secondary antibody (Ab Frontier, Seoul, Korea) in the blocking solution for 1 h at room temperature and followed by three times of rinsing with PBST (each for 10 min). The antigen-antibody complex on the bands was visualized using a chemiluminescence kit (Santa Cruz Biotechnology, Santa Cruz, CA). The relative band intensities of AmAChE1 and AmAChE2 in X-ray films were calculated using KODAK ID Image Analysis Software (Eastman Kodak Company, Rochester, NY) and used for quantification based on the observation that the band intensity is proportional to the log value of protein quantity (Fig. S7).

#### Statistical Analysis

SPSS for Windows 17.0 (IBM, Armonk, NY, USA) was used for data analysis. All of the data were presented as the mean ± SE. In the screen tent experiment, one-way ANOVA followed by Tukey’s test and linear regression were used to analyze the changes in the unsealed brood area in beehives after screen tent installation or elimination (Table 1).

## Additional Information

**How to cite this article:** Kim, Y. H. *et al*. Expression of acetylcholinesterase 1 is associated with brood rearing status in the honey bee, *Apis mellifera. Sci. Rep.* **7**, 39864; doi: 10.1038/srep39864 (2017).

**Publisher's note:** Springer Nature remains neutral with regard to jurisdictional claims in published maps and institutional affiliations.

## Supplementary Material

Supplementary Information

## Figures and Tables

**Table 1:** Correlation of changes of sealed brood area followed by screen tent installation and elimination.

	Estimated Coefficients ± SE	P value^a^
(+) Screen tent		
Sealed brood area	−0.015 ± 0.002	<0.0001
(−) Screen tent		
Sealed brood area	0.029 ± 0.002	<0.0001

^a^Linear regression analysis.

## References

[b1] Toutant J. P., Arpagaus M. & Fournier D. Native molecular forms of head acetylcholinesterase from adult *Drosophila melanogaster*-Quaternary structure and hydrophobic character. J Neurochem 50, 209–218 (1988). 3121787 10.1111/j.1471-4159.1988.tb13251.x

[b2] Kim Y. H. & Lee S. H. Which acetylcholinesterase functions as the main catalytic enzyme in the Class Insecta? Insect Biochem Mol Biol 43, 47–53 (2013). 23168079 10.1016/j.ibmb.2012.11.004

[b3] Huchard E. . Acetylcholinesterase genes within the Diptera: takeover and loss in true flies. Proc R Soc B Biol Sci 273, 2595–2604 (2006). 10.1098/rspb.2006.3621 PMC1635460 17002944

[b4] Weill M. . A novel acetylcholinesterase gene in mosquitoes codes for the insecticide target and is non-homologous to the *ace* gene in *Drosophila*. Proc Biol Sci 269, 2007–2016 (2002). 12396499 10.1098/rspb.2002.2122 PMC1691131

[b5] Fournier D., Berge J. B., Dealmeida M. L. C. & Bordier C. Acetylcholinesterases from *Musca domestica* and *Drosophila melanogaster* brain are linked to membranes by a glycophospholipid anchor sensitive to an endogenous phospholipase. J Neurochem 50, 1158–1163 (1988). 2831298 10.1111/j.1471-4159.1988.tb10587.x

[b6] Baek J. H. . Identification and characterization of *ace1*-type acetylcholinesterase likely associated with organophosphate resistance in *Plutella xylostella*. Pest Biochem Physiol 81, 164–175 (2005).

[b7] Lee D. W., Kim S. S., Shin S. W., Kim W. T. & Boo K. S. Molecular characterization of two acetylcholinesterase genes from the oriental tobacco budworm, *Helicoverpa assulta* (Guenee). Biochim Biophys Acta 1760, 125–133 (2006). 16352398 10.1016/j.bbagen.2005.10.009

[b8] Lee S. W. . Molecular characterization of two acetylcholinesterase cDNAs in *Pediculus* human lice. J Med Entomol 44, 72–79 (2007). 17294923 10.1603/0022-2585(2007)44[72:mcotac]2.0.co;2

[b9] Kim J. I., Jung C. S., Koh Y. H. & Lee S. H. Molecular, biochemical and histochemical characterization of two acetylcholinesterase cDNAs from the German cockroach *Blattella germanica*. Insect Mol Biol 15, 513–522 (2006). 16907838 10.1111/j.1365-2583.2006.00666.x

[b10] Seong K. M., Kim Y. H., Kwon D. H. & Lee S. H. Identification and characterization of three cholinesterases from the common bed bug, Cimex lectularius. Insect Mol Biol 21, 149–159 (2012). 22136067 10.1111/j.1365-2583.2011.01118.x

[b11] Nabeshima T. . An amino acid substitution attributable to insecticide-insensitivity of acetylcholinesterase in a Japanese encephalitis vector mosquito, *Culex tritaeniorhynchus*. Biochem Biophys Res Commun 313, 794–801 (2004). 14697262 10.1016/j.bbrc.2003.11.141

[b12] Weill M. . The unique mutation in *ace-1* giving high insecticide resistance is easily detectable in mosquito vectors. Insect Mol Biol 13, 1–7 (2004). 14728661 10.1111/j.1365-2583.2004.00452.x

[b13] Kim Y. H., Choi J. Y., Je Y. H., Koh Y. H. & Lee S. H. Functional analysis and molecular characterization of two acetylcholinesterases from the German cockroach, *Blattella germanica*. Insect Mol Biol 19, 765–776 (2010). 20738424 10.1111/j.1365-2583.2010.01036.x

[b14] Olivera S., Rodriguez-Ithurralde D. & Henley J. M. Acetylcholinesterase promotes neurite elongation, synapse formation, and surface expression of AMPA receptors in hippocampal neurones. Mol Cell Neurosci 23, 96–106 (2003). 12799140 10.1016/s1044-7431(03)00021-6 PMC3314531

[b15] Ballard C. G., Greig N. H., Guillozet-Bongaarts A. L., Enz A. & Darvesh S. Cholinesterases: roles in the brain during health and disease. Curr Alzheimer Res 2, 307–318 (2005). 15974896 10.2174/1567205054367838

[b16] Gauthier M., Belzunces L. P., Zaoujal A., Colin M. E. & Richard D. Modulatory effect of learning and memory on honey bee brain acetylcholinesterase activity. Comp Biochem Physiol C Pharmacol Toxicol Endocrinol 103, 91–95 (1992).

[b17] Shapira M., Thompson C. K., Soreq H. & Robinson G. E. Changes in neuronal acetylcholinesterase gene expression and division of labor in honey bee colonies. J Mol Neurosci 17, 1–12 (2001). 11665858 10.1385/JMN:17:1:1

[b18] Kang J. S., Lee D. W., Koh Y. H. & Lee S. H. A soluble acetylcholinesterase provides chemical defense against xenobiotics in the pinewood nematode. PLoS One 6, e19063 (2011). 21556353 10.1371/journal.pone.0019063 PMC3083410

[b19] Kim Y. H., Cha D. J., Jung J. W., Kwon H. W. & Lee S. H. Molecular and kinetic properties of two acetylcholinesterases from the Western honey bee, *Apis mellifera*. PLoS One 7, e48838 (2012). 23144990 10.1371/journal.pone.0048838 PMC3492254

[b20] Kim Y. H., Kwon D. H., Ahn H. M., Koh Y. H. & Lee S. H. Induction of soluble AChE expression via alternative splicing by chemical stress in *Drosophila melanogaster*. Insect Biochem Mol Biol 48, 75–82 (2014). 24637386 10.1016/j.ibmb.2014.03.001

[b21] Lu Y. H. . Cholinergic and non-cholinergic functions of two acetylcholinesterase genes revealed by gene-silencing in *Tribolium castaneum*. Sci Rep 2 (2012). 10.1038/srep00288 PMC3286809 22371826

[b22] Lu Y. H. . Genome organization, phylogenies, expression patterns, and three-dimensional protein models of two acetylcholinesterase genes from the red flour beetle. PLoS One 7 (2012). 10.1371/journal.pone.0032288 PMC3281121 22359679

[b23] Joshi N. C. & Joshi P. C. Foraging behaviour of Apis *Spp*. on apple flowers in a subtropical environment. N Y Sci J 3, 71–76 (2010).

[b24] Blaschon B., Guttenberger H., Hrassnigg N. & Crailsheim K. Impact of bad weather on the development of the broodnest and pollen stores in a honeybee colony (Hymenoptera: Apidae). Entomol Gen 24, 49–60 (1999).

[b25] Meshorer E. . Alternative splicing and neuritic mRNA translocation under long-term neuronal hypersensitivity. Science 295, 508–512 (2002). 11799248 10.1126/science.1066752

[b26] Haertl R., Gleinich A. & Zimmermann M. Dramatic increase in readthrough acetylcholinesterase in a cellular model of oxidative stress. J Neurochem 116, 1088–1096 (2011). 21198638 10.1111/j.1471-4159.2010.07164.x

[b27] Zimmerman G. & Soreq H. Readthrough acetylcholinesterase-A multifaceted inducer of stress reactions. J Mol Neurosci 30, 197–200 (2006). 17192675 10.1385/JMN:30:1:197

[b28] Birikh K. R., Sklan E. H., Shoham S. & Soreq H. Interaction of “readthrough” acetylcholinesterase with RACK1 and PKC beta II correlates with intensified fear-induced conflict behavior. Proc Natl Acad Sci USA 100, 283–288 (2003). 12509514 10.1073/pnas.0135647100 PMC140952

[b29] Garcia-Ayllon M. S., Millan C., Serra-Basante C., Bataller R. & Saez-Valero J. Readthrough acetylcholinesterase is increased in human liver cirrhosis. PLoS One 7 (2012). 10.1371/journal.pone.0044598 PMC3441564 23028565

[b30] Meshorer E. . SC35 promotes sustainable stress-induced alternative splicing of neuronal acetylcholinesterase mRNA. Mol Psychiatry 10, 985–997 (2005). 16116489 10.1038/sj.mp.4001735

[b31] Singh A., Jaiswal S. K. & Sharma B. Effect of low temperature stress on acetylcholinesterase activity and its kinetics in 5th instar larvae of *Philosamia ricini*. J Biochem Res 1, 17–25 (2013).

[b32] Seeley T. D. Adaptive significance of the age polyethism schedule in honeybee colonies. Behav Ecol Sociobiol 11, 287–293 (1982).

[b33] Schulz D. J. & Robinson G. E. Biogenic amines and division of labor in honey bee colonies: behaviorally related changes in the antennal lobes and age-related changes in the mushroom bodies. J Comp Physiol A Neuroethol Sens Neural Behav Physiol 184, 481–488 (1999). 10.1007/s003590050348 10377981

[b34] Withers G. S., Fahrbach S. E. & Robinson G. E. Selective neuroanatomical plasticity and division of labour in the honeybee. Nature 364, 238–240 (1993). 8321320 10.1038/364238a0

[b35] Stabentheiner A., Kovac H. & Brodschneider R. Honeybee colony thermoregulation-Regulatory mechanisms and contribution of individuals in dependence on age, location and thermal stress. PLoS One 5 (2010). 10.1371/journal.pone.0008967 PMC2813292 20126462

[b36] Toth A. L., Kantarovich S., Meisel A. F. & Robinson G. E. Nutritional status influences socially regulated foraging ontogeny in honey bees. J Exp Biol 208, 4641–4649 (2005). 16326945 10.1242/jeb.01956

[b37] Johnson B. R. Division of labor in honeybees: form, function, and proximate mechanisms. Behav Ecol Sociobiol 64, 305–316 (2010). 20119486 10.1007/s00265-009-0874-7 PMC2810364

[b38] Delaplane K. S., van der Steen J. & Guzman-Novoa E. Standard methods for estimating strength parameters of *Apis mellifera* colonies. J Apic Res 52 (2013).

[b39] Bradford M. M. A rapid and sensitive method for the quantitation of microgram quantities of protein utilizing the principle of protein-dye binding. Anal Biochem 72, 248–254 (1976). 942051 10.1016/0003-2697(76)90527-3

