# Supplementary material for: Expression of acetylcholinesterase 1 is associated with brood rearing status in the honey bee, Apis mellifera
Source: Sci Rep. 2017 Jan 3;7:39864. doi: 10.1038/srep39864 (PMC5206625; doi:10.1038/srep39864)
Supplement: Supplementary Information [file srep39864-s1.pdf]

Supplementary Information

Expression of Acetylcholinesterase 1 is associated with brood rearing status in the honey bee, *Apis mellifera*

Young Ho Kim[1,2,*], Ju Hyeon Kim[1,3], Kyungmun Kim[1] and Si Hyeock Lee[1,4,*]

[1]Department of Agricultural Biotechnology, Seoul National University, Seoul 151-921, Korea

[2]Department of Applied Biology, Kyungpook National University, Sangju 742-711, Korea

[3]Department of Veterinary and Animal Sciences, University of Massachusetts, Amherst, MA, USA

[4]Research Institute for Agriculture and Life Sciences, Seoul National University, Seoul, Korea

[*]Co-corresponding authors, yhkim05@knu.ac.kr (YHK); shlee22@snu.ac.kr (SHL)

a. Head

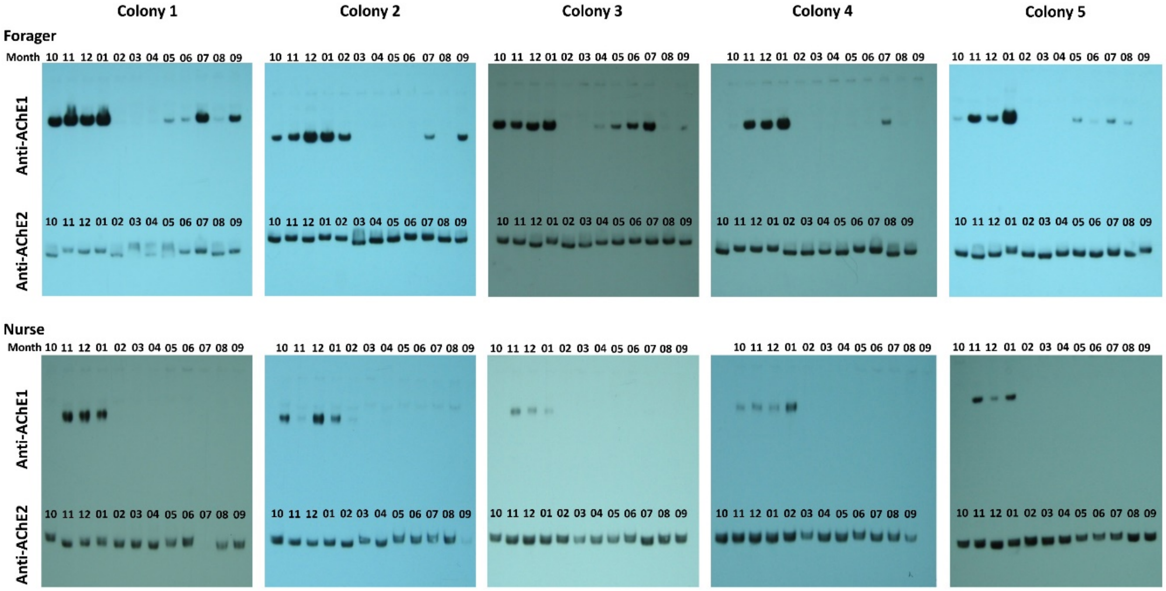

b. Abdomen

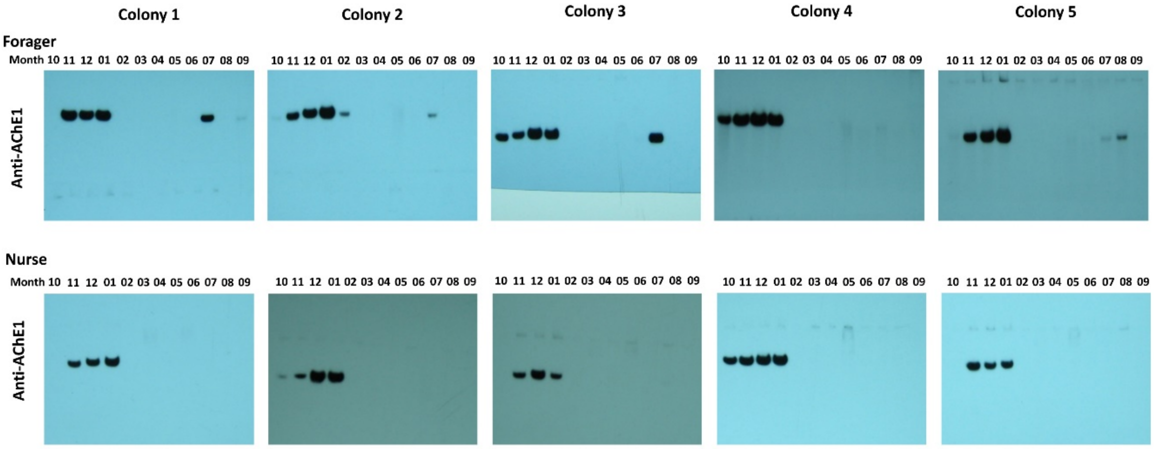

c. Temperature stress vs. AmAChE1 expression

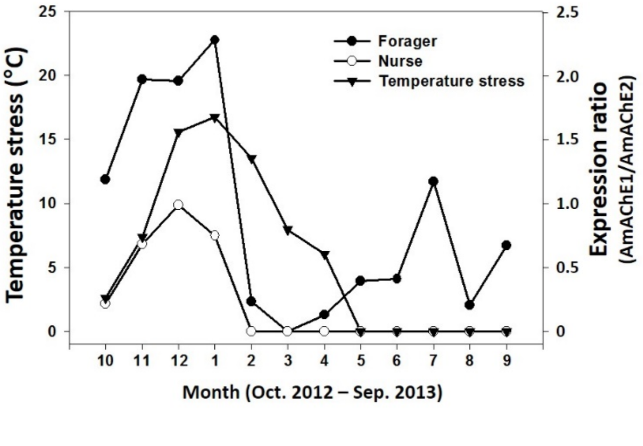

Supplementary Figure 1: Seasonal expression profiles of AmAChE1 and AmAChE2 (a, b) (from Figure 1a). Comparison of the seasonal expression patterns of AmAChE1 and temperature stress (c). Honey bee samples were collected from 5 different colonies over one year. Protein samples extracted from the heads (a) and abdomens (b) of foragers and nurses were detected by Western blotting using anti-AmAChE1 and anti-AmAChE2. The expression rates of AmAChE1 originated from Fig. 1. Temperature stress was calculated from the difference between the recorded average temperatures of each month and 16°C, which is a threshold temperature for foraging[23] (c).

a. Field to Greenhouse

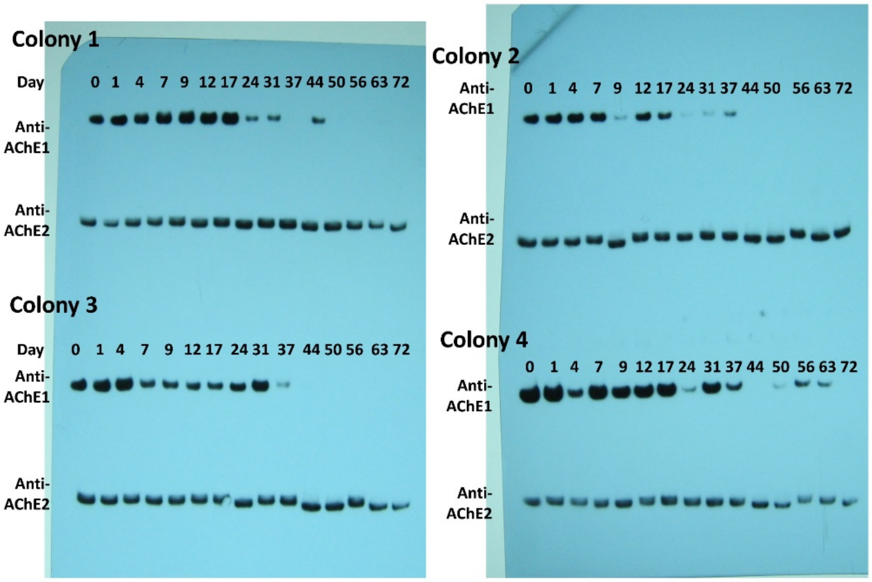

b. Greenhouse to Field

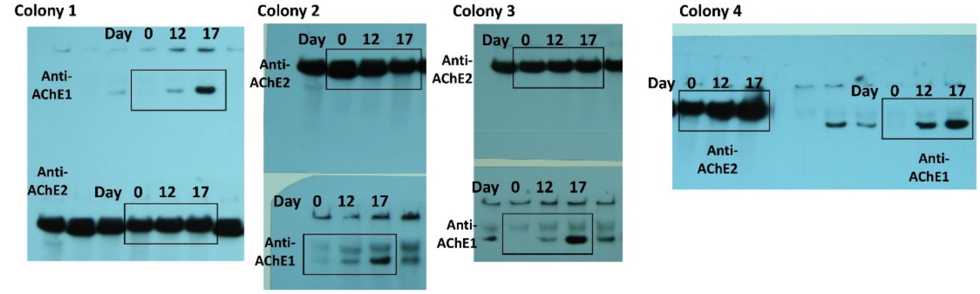

c. Recorded temperature in the green houses and field

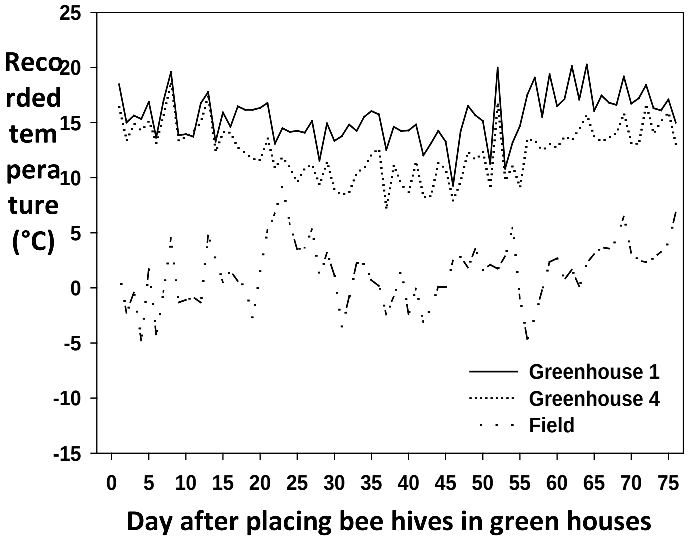

Supplementary Figure 2: Western blot analysis of the expression patterns of two AmAChEs of overwintering honey bees following placement in a greenhouse (a) (from Figure 2a) and field (b) (from Figure 2b). Recorded temperature in the green houses and field (c). Overwintering beehives were placed in a greenhouse for 72 days to allow for brood rearing in winter (a). The beehives were moved back to the field to suppress brood rearing (b). Temperature information was recorded by a data logger in the two greenhouses and field (c).

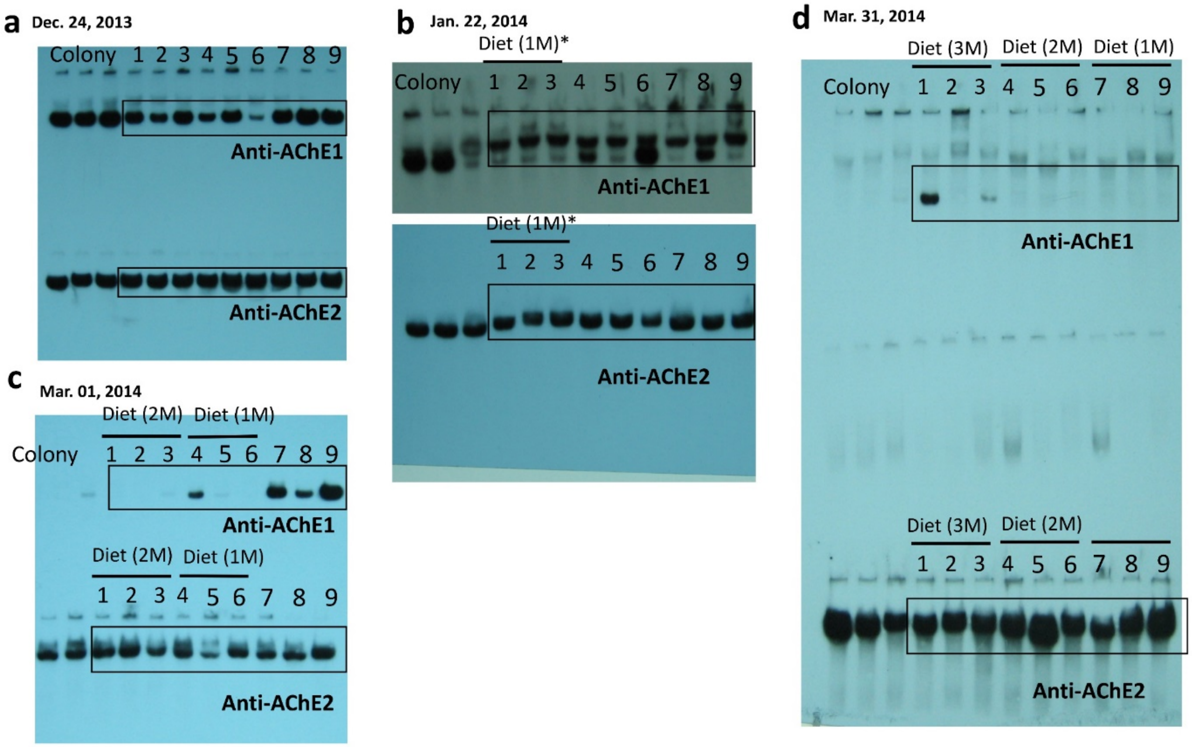

Supplementary Figure 3: Western blot analysis of expression of AmAChE induced by supplementing with an artificial diet (from Figure 3). Protein samples were prepared from nine overwintering beehives every month from Dec. 24, 2013 to Mar. 31, 2014. On Dec. 24, 2013, an artificial diet was initially supplied to Colonies 1, 2 and 3 (a). Colonies 1 to 6 and all of the colonies were supplied with the artificial diet on Jan. 22, 2014 (b) and Mar. 01, 2014 (c), respectively. A month later (on Mar. 31, 2014), protein samples, prepared every month, were separated in a native-PAGE gel, and two AChEs were detected by Western blotting. Asterisks indicate the period of artificial diet supplementation.

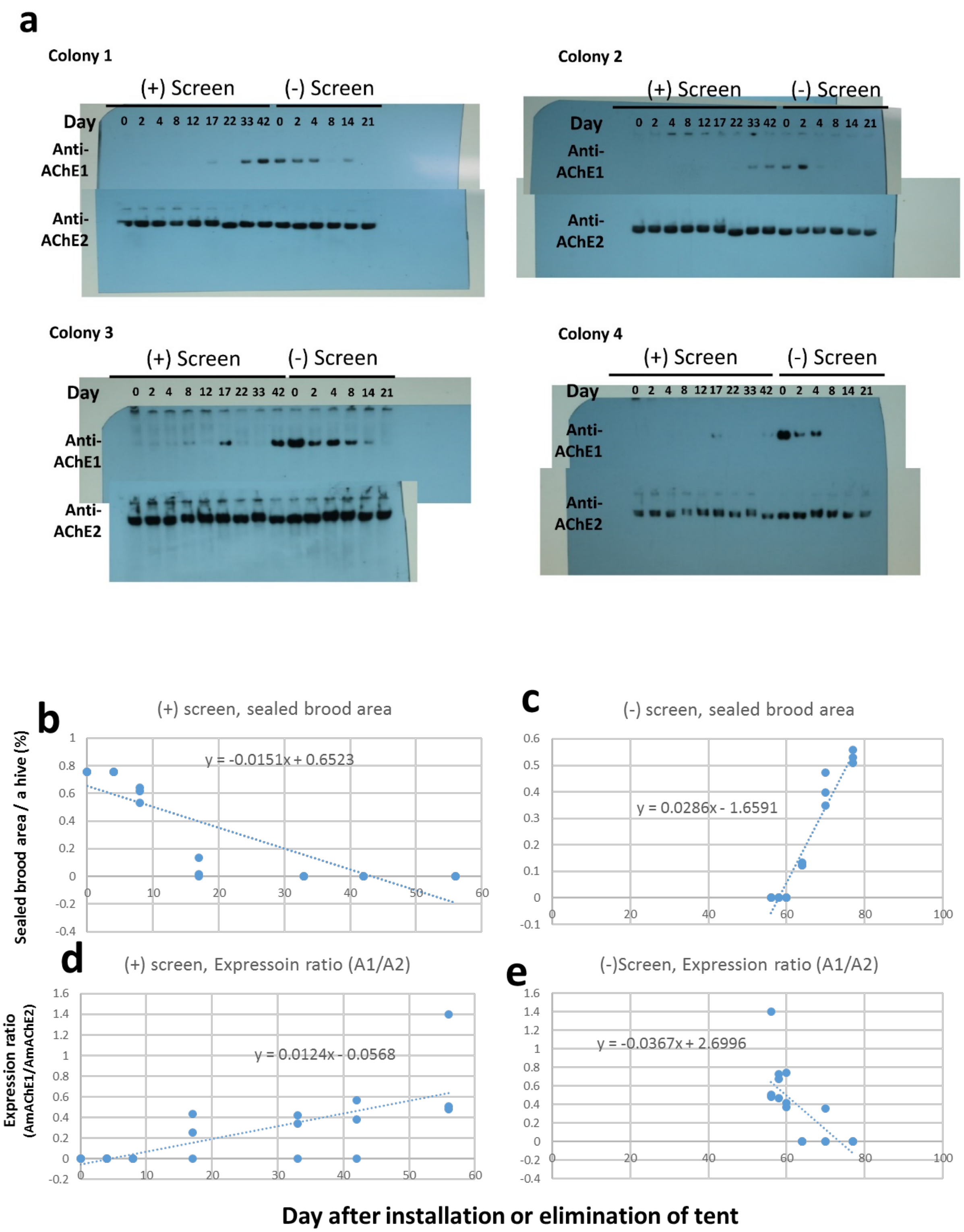
a
Colony 1
(+) Screen
(-) Screen
Day
0 2 4 8 12 17 22 33 42 0 2 4 8 14 21
Anti-AChE1
Anti-AChE2
Colony 2
Colony 3
Colony 4
b
(+) screen, sealed brood area
y = -0.0151x + 0.6523
Sealed brood area / a hive (%)
c
(-) screen, sealed brood area
y = 0.0286x - 1.6591
d
(+) screen, Expressoin ratio (A1/A2)
y = 0.0124x - 0.0568
Expression ratio (AmAChE1/AmAChE2)
e
(-)Screen, Expression ratio (A1/A2)
y = -0.0367x + 2.6996
Day after installation or elimination of tent

Supplementary Figure 4: Western blot analysis of the expression patterns of two AmAChEs of honey bees from four active beehives (a) and a correlation analysis of the changes of brood rearing area (b and c) and AmAChE1 expression (d and e) following screen tent installation and elimination.

Four colonies exhibiting high brood-rearing activity were selected in late spring and placed in a screen tent to artificially suppress brood rearing by blocking foraging. At 56 days post-foraging inhibition, beehives were allowed to restore brood rearing by removing the screen. Two AmAChEs were detected by Western blotting (a). The sealed brood area decreased (b) and increased (c) by screen tent installation and elimination, respectively, and was statistically measured by linear regression analysis ($P<0.0001$).

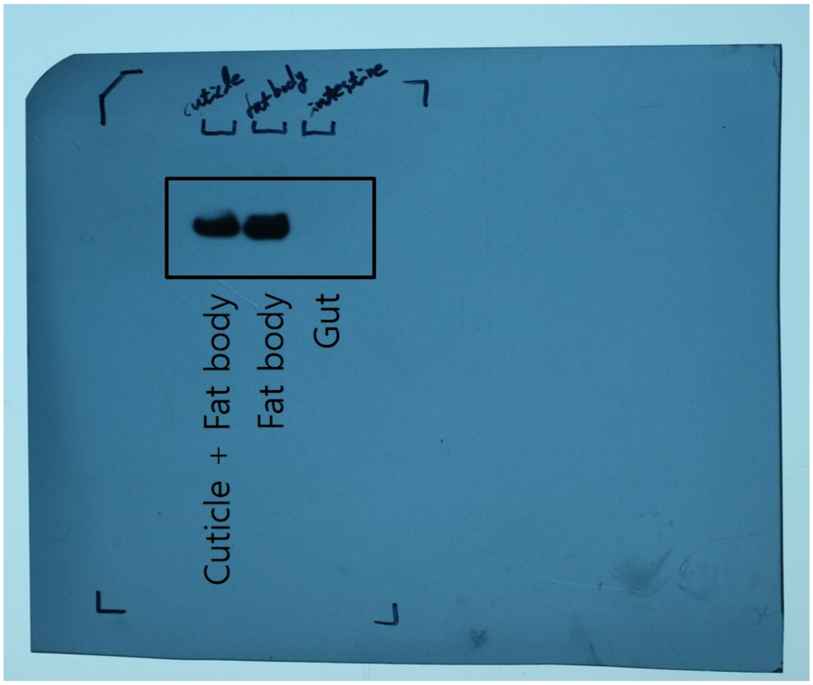

Supplementary Figure 5: **Western blot analysis of the expression of AmAChE1 in different tissues of foragers.** Honey bee abdomen was dissected and gut tissues (alimentary tract, Malpighian tubules, etc.) were isolated first (Gut). Then, fat body tissues were isolated from the inside of integument by scraping (Fat body). The integument with remaining fat body tissues attached was also used for protein extraction (Cuticle + Fat body). Protein was extracted from these three tissue samples (Cuticle + Fat body, Fat body and Gut) with 0.1 M Tris-HCl (pH 7.8) containing 0.5% Triton X-100. Protein samples (8 µg) were separated by native-PAGE and Western blotting was conducted using anti-AChE1 antibody.

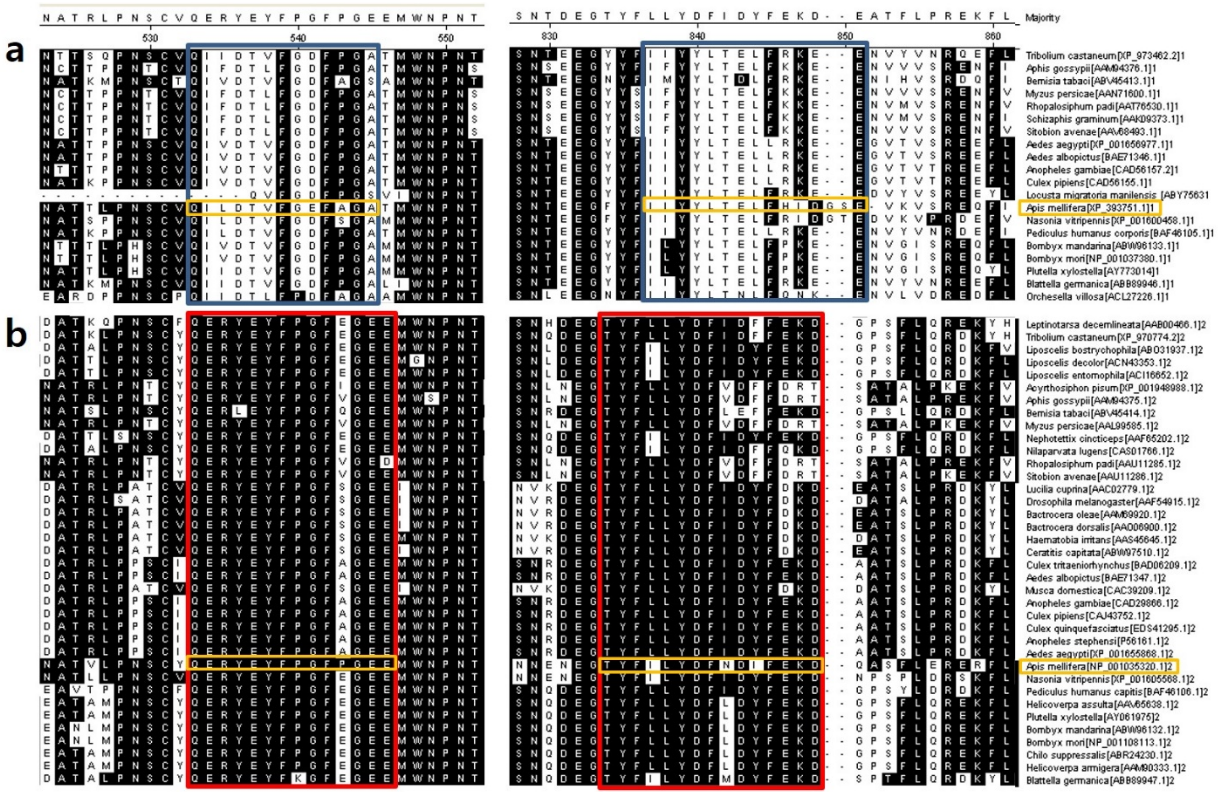

Supplementary Figure 6: Multiple alignments of insect AChE amino acid sequences from 20 AChE1 and 37 AChE2 genes used to identify suitable AChE1- and AChE2-specific antigen regions. Two regions specific to each AChE were designed (a for AChE1 vs. b for AChE2). Yellow boxes indicate the amino acid sequences of AmAChE1 and AChE2. GenBank accession numbers for AChEs are shown in square brackets (modified from Kim and Lee, 2013).

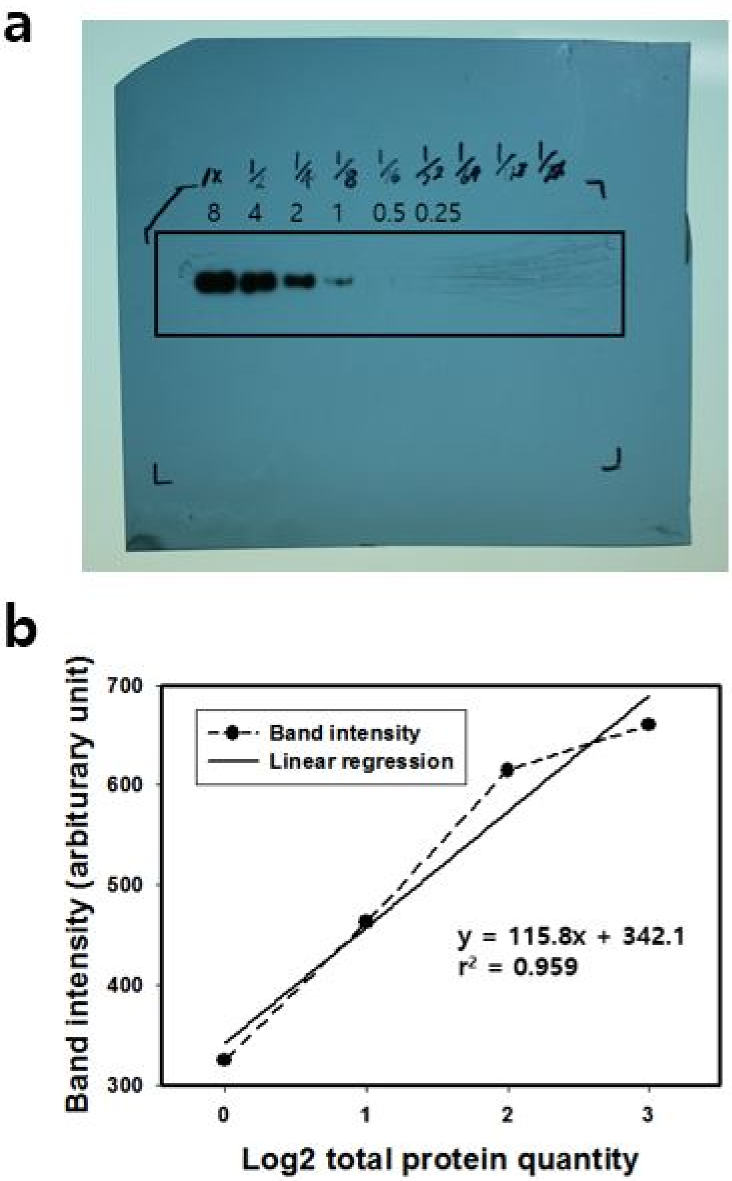

Supplementary Figure 7: Serial dilution and detection of honey bee protein (a) and linear relationship between the band intensities and log values of protein quantity (b). The series of two-fold diluted protein sample (8, 4, 2, 1, ~ 0.031 μg) extracted from forager head were separated by native-PAGE, and AmAChE1 was detected with Anti-AChE1 antibody (a). Then, band intensities were measured using an image analysis software and plotted against log values of protein quantities (b).
